# Superparamagnetic Iron Oxide Particles (VSOPs) Show Genotoxic Effects but No Functional Impact on Human Adipose Tissue-Derived Stromal Cells (ASCs)

**DOI:** 10.3390/ma14020263

**Published:** 2021-01-07

**Authors:** Katrin Radeloff, Mario Ramos Tirado, Daniel Haddad, Kathrin Breuer, Jana Müller, Sabine Hochmuth, Stephan Hackenberg, Agmal Scherzad, Norbert Kleinsasser, Andreas Radeloff

**Affiliations:** 1 Department of Otorhinolaryngology, Head and Neck Surgery, University of Oldenburg, 26122 Oldenburg, Germany; j.a.mueller@uni-oldenburg.de (J.M.); sabine.hochmuth@uni-oldenburg.de (S.H.); andreas.radeloff@uni-oldenburg.de (A.R.); 2 Department of Otorhinolaryngology, Plastic, Aesthetic and Reconstructive Head and Neck Surgery, University of Wuerzburg, 97080 Wuerzburg, Germany; mramos76@yahoo.com (M.R.T.); hackenberg_s@ukw.de (S.H.); scherzad_a@ukw.de (A.S.); kleinsasse_n@ukw.de (N.K.); 3 Fraunhofer Development Center X-ray Technology EZRT, Department Magnetic Resonance and X-ray Imaging, A Division of Fraunhofer Institute for Integrated Circuits IIS, 97074 Wuerzburg, Germany; daniel.haddad@iis.fraunhofer.de; 4 Department of Radiation Oncology, University of Wuerzburg, 97080 Wuerzburg, Germany; Breuer_K@ukw.de

**Keywords:** ASCs, adipose tissue-derived stromal cells, VSOP, iron oxide nanoparticles, toxicity, MRI, cell labeling

## Abstract

Adipose tissue-derived stromal cells (ASCs) represent a capable source for cell-based therapeutic approaches. For monitoring a cell-based application in vivo, magnetic resonance imaging (MRI) of cells labeled with iron oxide particles is a common method. It is the aim of the present study to analyze potential DNA damage, cytotoxicity and impairment of functional properties of human (h)ASCs after labeling with citrate-coated very small superparamagnetic iron oxide particles (VSOPs). Cytotoxic as well as genotoxic effects of the labeling procedure were measured in labeled and unlabeled hASCs using the MTT assay, comet assay and chromosomal aberration test. Trilineage differentiation was performed to evaluate an impairment of the differentiation potential due to the particles. Proliferation as well as migration capability were analyzed after the labeling procedure. Furthermore, the labeling of the hASCs was confirmed by Prussian blue staining, transmission electron microscopy (TEM) and high-resolution MRI. Below the concentration of 0.6 mM, which was used for the procedure, no evidence of genotoxic effects was found. At 0.6 mM, 1 mM as well as 1.5 mM, an increase in the number of chromosomal aberrations was determined. Cytotoxic effects were not observed at any concentration. Proliferation, migration capability and differentiation potential were also not affected by the procedure. Labeling with VSOPs is a useful labeling method for hASCs that does not affect their proliferation, migration and differentiation potential. Despite the absence of cytotoxicity, however, indications of genotoxic effects have been demonstrated.

## 1. Introduction

Adipose tissue-derived stromal cells (ASCs) share, as a subtype of mesenchymal stem cells (MSCs), their characteristic properties. Thus, they are a valuable instrument for cell-based therapeutic approaches in tissue regeneration and they are known for useful effects on damaged tissue, such as the promotion of wound healing and prevention of scarring [1,2,3,4]. Due to their promotion of neo-vascularization, ASCs also improve the long-term survival and volume stability of transplanted fat grafts when added to the grafts as so-called cell-assisted lipotransfer (CAL) [5,6,7,8]. The beneficial effects of ASCs are not based primarily on their multi-lineage differentiation potential into tissue-specific cells but on paracrine secretion of various trophic factors, such as growth factors, cytokines, exosomes and extracellular microvesicles. These factors activate resident and circulating cells, promote neovascularization and show antiapoptotic and immunomodulatory effects [3,9,10,11,12,13,14,15,16,17]. In addition to their promising properties for regenerative medicine, human (h) ASCs can be harvested in large numbers without relevant donor-site morbidity [18].

In vivo visualization of magnetically labeled cells by magnetic resonance imaging (MRI) is a commonly used method to monitor cell distribution and the success as well as safety of cell therapy [9,19,20,21,22,23,24]. In addition, the labeling of the transplanted cells makes it possible to distinguish them from the resident cells [19,21,23,25,26,27,28,29]. Therefore, iron oxide nanoparticles (IONPs) are used for labeling various cell types: ASCs, mesenchymal stem cells from the bone marrow (BMSCs), chondrocytes [19,21,23,30,31], human adult neural stem cells (haNCSs) [31], murine embryonic stem cells (mESCs) [31] and human umbilical blood mononuclear cells (hUCBCs) [27]. In addition, they are applied in cancer imaging and treatment and used for targeted drug and gene delivery [24,29,32,33,34]. Different types of IONPs with various surface coating materials and particle sizes are available, such as citrate-coated very small superparamagnetic iron oxide particles (VSOPs) [19,20,23,33,35,36,37,38].

VSOPs (C200) have a small diameter of about 11 nm and a negative surface charge, which enables an efficient uptake into the cells without the necessity of a transfection agent [23]. In addition, the functionality of MSCs should be less affected than with other IONPs due to the small diameter of the VSOPs [19]. VSOPs are metabolized in iron metabolic pathways [19,31] and are supposed to have a high biocompatibility, although adverse impacts of degradation products on cell physiology and functions cannot be excluded [19]. Signal loss and hypointense, dark spots show present VSOPs-labeled cells in the MRI images [19,31]. Labeling of cells with these so-called “negative” MRI contrast agents [35] results in reduction in the T2 relaxation time, followed by hypointensity, i.e., signal darkening in T_2_*-weighted images [23,31,39].

The extension of the scope of IONPs leads to an increasing exposure of humans to these particles [36,40]. Recently, Gaharwar et al. investigated the biodistribution and accumulation of intravenously applied IONPs in the organs of rats. They showed a dose-dependent accumulation of IONPs and ultrastructural changes with tissue damage [40]. However, the application of IONPs or the labeling of cells with IONPs in cell-based therapies should not lead to a change in cell properties and functions or cause lasting damage. It was described in previous studies that IONPs can potentially induce toxic effects due to the production of reactive oxygen species (ROS), cell membrane leakage, DNA damage, cell cycle alterations, the influence on the integrity of mitochondria, promotion of cell apoptosis as well as the alteration of gene expression in exposed human cells [20,24,39,41,42,43,44]. Due to the different cell types and different IONPs used in the studies, the results in the literature are inconsistent with regard to toxic effects and functional impairment of the labeled cells, which makes a direct comparison of the published data difficult [19,21,30,43,44,45,46,47,48,49,50,51,52].

Human ASCs labeled with VSOPs were used for the analyses in the present study to evaluate potential cyto- and genotoxic effects of the labeling procedure. Furthermore, an impairment of the migration and proliferation capacity as well as the multilineage differentiation capacity of the hASCs by the labeling procedure were assessed. The results should provide additional information on the interaction between VSOPs commonly used for cell labeling and hASCs.

## 2. Material and Methods

### 2.1. Isolation and Expansion of Human Adipose Tissue-Derived Stromal Cells (hASCs)

Human ASCs were isolated from liposuction material as described several times before [53,54]. This study was approved by the Institutional Review Board of the University Hospital Wuerzburg (grant #72/06). Informed consent was obtained from every patient.

The liposuction material was first washed with phosphate-buffered saline solution (PBS; Roche Diagnostics, Mannheim, Germany) containing 1% penicillin/streptomycin (P/S; Biochrom AG, Berlin, Germany), followed by a digestion step with collagenase P (Roche Diagnostics) under continuous shaking. After centrifugation and discarding the supernatant, erythrocyte lysis buffer was added. Following an additional centrifugation and washing step, the cell pellet was dissolved in expansion medium (EM-DMEM), which consisted of Dulbecco’s modified Eagle’s medium (Gibco Invitrogen, Karlsruhe, Germany) plus 1% P/S and 10% fetal calf serum (FCS; Linaris, Wertheim-Bettingen, Germany). Cultivation was performed at 37 °C in a humidified atmosphere and 5% CO_2_ until the cells reached a confluence of 80%. Cells were then detached with 0.25% trypsin plus 1 mM EDTA (Gibco Invitrogen) and frozen. Therefore, 1 × 10^6^ hASCs were resuspended in 1 mL cryopreservation medium (80% FCS, 10% DMEM and 10% dimethylsulfoxide (DMSO, Sigma-Aldrich Chemie GmbH, Taufkirchen, Germany)), frozen at −80 °C in an ethanol-jacketed closed container for 2 days and afterwards stored in liquid nitrogen.

For the investigations, the hASCs of a total of 6 patients were thawed very quickly and centrifuged to remove DMSO from the cryopreservation medium. Subsequently, the hASCs were cultivated in EM-DMEM. ASCs of passages 2 and 3 were used for the investigations of the present study.

### 2.2. Labeling of hASCs with VSOPs

Citrate-coated VSOPs C200 from Ferropharm (Teltow, Germany) with an iron oxide core of 5 nm were used for the analyses [19,31,35]. According to the manufacturer, the roundish particles are between 8 and 11 nm in size. In a recently published study of our own group using the same VSOPs for long-term analyses, the dynamic light scattering obtained a z-average hydrodynamical diameter of 16.47 nm, and a polydispersity index of 0.282 was yielded. The zeta potential at pH 7.4 was −28 mV with an isoelectric point of 3.2 [55]. The information on relaxivity of VSOPs C200 was taken from the publication of Stroh et al. [39]: R1 22.5 mmol^−1^ s^−1^ at 0.47 T and R2 49.7 mmol^−1^ s^−1^ at 0.47.

The labeling procedure was carried out as described previously, based on the manufacturer’s specifications [9,19]. VSOPs were added to the EM-DMEM in concentrations of 1.5 mM, 1 mM, 0.6 mM, 0.3 mM, 0.15 mM and 0.015 mM. The hASCs were then incubated for 90 min at 37 °C and 5% CO_2_. Afterwards, the hASCs were washed three times with 1xPBS. After 24 h the cells were detached and used for the experiments.

#### 2.2.1. Detection of VSOPs-Labeled hASCs with Prussian Blue Staining and TEM

The Prussian blue staining was used to detect intracellular VSOPs. Human ASCs were seeded on slides, fixed and incubated with 1% potassium ferrocyanide in 1% hydrochloric acid. After washing the cells, they were counterstained with nuclear fast red [56].

The intracellular particle distribution and accumulation of VSOPs was visualized by transmission electron microscopy (TEM), as described previously [55,57]. VSOPs-labeled and unlabeled hASCs were fixed in 0.1 M sodium cacodylate buffer (pH 7.2) with 2.5% glutaraldehyde and 2% formaldehyde. The cells were postfixed with 2% osmium tetroxide in 50 mM sodium cacodylate buffer (pH 7.2). After washing the samples, the staining was performed overnight with 0.5% aqueous uranyl acetate. Following dehydration and embedding in epoxy resion (Epon 812), the samples were cut into ultra-thin sections of 60 nm thickness and examined with the transmission electron microscope. The investigations were kindly performed by the group of Prof. Dr. Krohne, Division of Electron Microscopy, Theodor-Boveri-Institut of the University of Wuerzburg. A Zeiss transmission electron microscope EM 900 (Carl Zeiss, Oberkochen, Germany) was used.

#### 2.2.2. High-Resolution MR Imaging (MRI)

MR imaging studies of agarose gel phantoms seeded with VSOPs-labeled hASCs were performed at 11.7 Tesla (T) with a maximum gradient strength of 0.66 T/m on a 500 MHz Bruker Avance 500 MRI system (Bruker BioSpin GmbH, Rheinstetten, Germany). The examinations were kindly performed at the Research Center Magnetic Resonance Bavaria, now Magnetic Resonance and X-ray Imaging Department, Development Center X-ray Technology EZRT, Fraunhofer Institute for Integrated Circuits IIS in Wuerzburg.

The hASCs were labeled with different concentrations of VSOPs between 0.15 mM and 1.5 mM for these studies to qualitatively evaluate the labeling efficacy. Labeled hASCs were resuspended in 1.5% agarose solution. The cell density in the gels was 1 × 10^6^ per mL. After solidification of the gels and subsequent fixation in 4% paraformaldehyde solution, the constructs were transferred into a 20 mm MRI tube, and placed within a ^1^H quadrature birdcage for MR imaging.

The images were acquired using a 3D FLASH sequence with 4 averages and the following parameters: echo time TE = 6 ms, repetition time TR = 100 ms, and field-of-view FOV = 25 × 20 × 20 mm^3^. The nominal spatial resolution was 98 × 78 × 78 µm^3^. A zero filling by a factor of two was applied in every spatial dimension during the image post processing.

### 2.3. Genotoxicity

#### 2.3.1. Alkaline Single-Cell Microgel Electrophoresis (Comet) Assay

As already described [53,57,58], the comet assay was used for the detection of DNA strand breaks and incomplete excision repair sites. Labeled and unlabeled hASCs were used for this purpose. A DMLB fluorescence microscope (Leica Microsystems, Wetzlar, Germany) with a filter system comprising a green excitation filter (515–560 nm), a dichromatic beamsplitter (580 nm) and an emission filter (590 nm) 400× magnification was used for evaluation. Unlabeled hASCs were treated with 200 µM directly alkylating methyl methanesulfonate (MMS; Sigma-Aldrich Chemie GmbH) to serve as positive controls. Two slides with 50 cells each (100 cells in total) for each patient and each VSOPs concentration were counted. The following parameters were analyzed to quantify DNA fragmentation using the COMET 5.5 image system (Kinetic Imaging, Liverpool, UK): tail DNA (TD), tail length (TL) and the product of the median migration distance and the percentage of DNA in the tail, the so-called olive tail moment (OTM) [59]. Following the recommendations of Tice et al. [58], the statistical analysis was based on the OTM values.

#### 2.3.2. Chromosomal Aberration (CA) Test

Human ASCs of passage 2 were used for the chromosome aberration test to exclude passage-dependent chromosome aberrations [54]. ASCs treated with 200 µM directly alkylating MMS served as positive controls. Chromosome sample preparation was based on a modification of the Sonoda protocol [60], as described previously [54]. To arrest cells in metaphase, 0.1 µg/mL colcemide (Gibco Invitrogen) was added to EM-DMEM for 1.5 h. After treatment with 0.4% KCl in PBS for 20 min at 37 °C, the hASCs were fixed in a freshly prepared Carnoy solution (3:1 mixture of methanol and acetic acid).

The cells were fixed twice at −20 °C before trypsinization. After seeding the cells on glass slides, the slides were placed on a heating plate (Zipperer GmbH, Staufen, Germany) at 90 °C for 30 min to dry before they were stored overnight at 60 °C. On the other day, the slides were incubated with 1% trypsin in PBS for 30 s and stained with 3% Giemsa solution (Merck Biosciences, Darmstadt, Germany, pH 6.4), then rinsed and air-dried. A competent analyst assessed the Giemsa-stained metaphase cells with an inverted light microscope for CA. A 100-fold magnification was used to evaluate CA. Fifty metaphase cells of each VSOPs concentration, unlabeled hASCs and positive controls, resulting in 300 metaphase cells per patient, were observed and CAs such as gaps, chromatid breaks, chromosome breaks, reunions as well as numerical aberrations were recorded according to the International System for Human Cytogenetic Nomenclature (ISCN) [61]. The ISCN defines a gap as a clear, non-staining region on a chromosome, and a break as a chromosomal discontinuity.

### 2.4. Cell Viability and Proliferation

#### 2.4.1. Trypan Blue Exclusion Test

After cell detachment, the cell viability of VSOPs-labeled and unlabeled hASCs was determined using the trypan blue exclusion test [62] to ensure an adequate number of viable cells before performing the comet assay. In principle, viable cells can exclude the dye and are transparent, whereas non-viable cells stain blue due to defects in the cell membrane and subsequent accumulation of the dye within the cell. A Neubauer chamber was used for cell counting. The percentage of viable cells was determined in 16 fields.

#### 2.4.2. MTT Assay

To evaluate the effect on the proliferation ability of hASCs and possible cytotoxic effects after VSOP labeling, the MTT (3-(4,5-dimethylthiazol-2-yl)-2,5-diphenyl tetrazolium bromide) test was performed [63]. Firstly, 1 × 10^4^ hASCs per well were seeded in a 96-well plate and incubated for 24 h with EM-DMEM at 37 °C in a 5% CO_2_ atmosphere. After 24 h, the hASCs were labeled with 1.5 mM and 0.015 mM VSOPs for 90 min, according to the protocol described above. Unlabeled hASCs were used as negative and cells treated with tert-butylhydroperoxide (t-BHP; Luperox^®^ TBH70X; Sigma-Aldrich Chemie GmbH) as positive controls. T-BHP induces cell apoptosis [64] and is routinely used by our study group. For each patient, concentration and time point, 8 wells were seeded. The analyses were performed immediately after labeling and after 24 h, 48 h and 72 h. After removing the medium and washing the cells, 20 µL (0.5 mg per mL medium) of MTT solution was added to each well, followed by incubation at 37 °C in a 5% CO_2_ atmosphere for 4 h. The medium was then replaced by 100 µL isopropanol. After 30 min, the color conversion of the blue formazan dye was measured at 570 nm with a multiplate reader (Titertek Multiscan PLUS (MKII), Pforzheim, Germany). The mean extinction values of 8 wells per patient, concentration and time point were used for the analysis. The values of untreated hASCs were adjusted to 100% viability. The viability of VSOPs-labeled cells and positive controls was expressed as a percentage of the unlabeled controls.

#### 2.4.3. Proliferation Analysis

VSOPs-labeled and unlabeled hASCs were seeded in 24-well plates with a density of 2.5 × 10^4^ cells each and maintained in EM-DMEM at 37 °C with 5% CO_2_ for 12 days. VSOPs concentrations of 1.5 mM and 0.15 mM were used for labeling. The proliferation analysis was performed in duplicate for each patient, each VSOPs concentration and for unlabeled cells, resulting in 10 wells per patient per day. Every second day the cells were detached with trypsin and counted with an automated cell counter (Casy^®^ Technologies, Innovatis AG, Reutlingen, Germany).

### 2.5. Scratch Assay for Wound Healing

The wound healing (scratch) assay was used to evaluate the migration capacity of VSOPs-labeled and unlabeled hASCs. The hASCs were labeled with 1.5 mM VSOP for 90 min. Labeled and unlabeled hASCs were seeded at a density of 1.5 × 10^5^ cells/well in 6-well plates. They were maintained in EM-DMEM in monolayer culture. After 24 h, a straight line wound was induced and cell debris was removed by washing with PBS. Wound width (time point 0 h) was measured after photography (Leica Microsystems) using ImageJ software. After a further incubation for 24 h, the wound width (time point 24 h) was measured and photographed. To show the relative migration of the cells, the images of both points of time were compared and “wound closure” was analyzed in relation to the percentage difference of the wound area at time 0 h. Analyses of the images for labeled and unlabeled hASCs were performed in triplicates for each patient under blinded conditions.

### 2.6. Multilineage Differentiation Potential

Adipogenic, osteogenic and chondrogenic differentiation were performed to evaluate the multidifferentiation potential and a possible impact of VSOPs labeling. The hASCs were labeled with 1.5 mM VSOPs, the highest concentration used for the experiments. In the literature, an impairment of the differentiation potential of MSCs as a function of IONPs concentration is described [51,52]. Defined media were used to induce differentiation. For adipogenic differentiation, EM-DMEM supplemented with 10 µM dexamethasone, 1 µg/mL insulin, 100 µM indomethacin and 500 µM 1-methyl-3-isobutylxanthine was used based on a modification [65] of the protocol of Pittenger et al. [66]. The osteogenic differentiation medium consists of EM-DMEM with 100 nM dexamethasone, 10 mM ß-glycerophosphate and 50 µg/mL ascorbic acid according to Jaiswal et al. [67]. Chondrogenic differentiation was induced with DMEM plus 1% P/S and 100 nM dexamethasone, 100 µg/mL sodium pyruvate, 50 µg/mL ascorbate-2-phosphate, 40 µg/mL proline, ITS-plus (Sigma-Aldrich Chemie GmbH) and 10 ng/mL TGF-ß3 (LONZA, Basel, Switzerland).

#### 2.6.1. Histology

In this study, 2 × 10^4^ VSOPs-labeled and unlabeled hASCs per well were plated in 4-wells (Greiner Bio-One GmbH, Frickenhausen, Germany) for adipogenic and osteogenic differentiation. The cells were cultivated for two and three weeks, respectively, and the medium was changed every other day. The chondrogenic differentiation was performed in a high-density three-dimensional pellet culture modified by Johnstone et al. [68]. Then, 3 × 10^5^ VSOPs-labeled and unlabeled hASCs were centrifuged in 15 mL polypropylene tubes (Cellstar, Greiner Bio-One) to form cell aggregates. The pellets were cultivated for three weeks and the medium was changed every other day. After three weeks, the pellets were fixed with 4% paraformaldehyde, washed and embedded using Tissue-Tek^®^ O.C.T™ Compound (Optimal Cutting Temperature Paraffin; Sakura, Alphen aan den Rijn, The Netherlands) for the frozen sections.

The Oil Red O stain was used to visualize intracellular lipid droplets after adipogenic differentiation. The presence of black nodules due to extracellular mineral deposition was detected by von Kossa staining after osteogenic differentiation. Extracellular deposition of calcium was also shown in red using the Alizarin Red staining. Chondrogenic differentiation was confirmed by the typical turquoise alcian blue staining of extracellular glycosaminoglycans.

ASCs from the same donor, in monolayer culture or as pellets were maintained in EM-DMEM for two weeks to serve as negative controls for the differentiation procedures.

#### 2.6.2. Real Time-PCR Analyses

The adipogenic, osteogenic and chondrogenic differentiation were quantified by Real-Time PCR analyses. Firstly, 1 × 10^5^ cells labeled and unlabeled hASCs per well were seeded in 6-wells (Greiner Bio-One GmbH) for adipogenic and osteogenic differentiation, while a 3D pellet culture was used for chondrogenic differentiation, as described above. As mentioned above, the samples were harvested and examined after 14 and 21 days, respectively. The RNeasy Mini Kit (Qiagen, Hilden, Germany) was used for the extraction of total-RNA, the High Capacity RNA-to-cDNA Master Mix (Applied Biosystems, Darmstadt, Germany) for reverse transcription. Real-Time PCR was performed in duplicates using 50 ng cDNA per replicate with standard Taqman^®^ assays on a Real-time PCR device (Applied Biosystems).

The gene expression of specific marker genes was quantified. For adipogenically differentiated samples, the fatty acid binding protein 4 (aP2; NM_001442.2), lipoproteinlipase (LPL; NM_000237.2) and leptin (NM_002303.5) were measured. The alkaline phosphatase (ALP; NM_000478.4), the bone gamma-carboxylglutamate protein (osteocalcin; NM_199173.4) and Runt-related transcription factor 2 (Runx-2/cbfa-1; NM_004348.3) were measured as osteogenic markers and aggrecan (NM_001135.3), the transcription factor SOX-9 (NM_000337.1) and collagen II (COL2A1; NM_033150.2) were used as chondrogenic marker genes. The ∆CT values were normalized to the expression of GAPDH (housekeeping gene, NM_002046.3). The RNA from fat tissue, cartilage and bone remaining after elective surgery was used as a positive control for the expression of the specific marker genes.

### 2.7. Statistical Analyses

For the statistical analyses and the graphs, GraphPad 8 (Graphpad Software, La Jolla, CA, USA) was used. For the analysis of differences in chromosomal aberrations, cell viability and DNA fragmentation (comet assay), a one-way ANOVA was applied. For the analysis of proliferation and migration capacity as well as to compare tissue specific marker gene expression, the unpaired t-test was used in case of Gaussian distribution, otherwise the Mann–Whitney U test was performed. A two-way ANOVA with Bonferroni post tests was performed for the MTT assay analysis.

Significance was indicated in the figures by asterisks and assumed for *p* < 0.05. Results were mostly charted as Boxplots with the box showing the median, the 1st quartile and the 3rd quartile. The whiskers present the minimal and maximal values. The columns show the mean and standard error of the mean (SEM).

## 3. Results

### 3.1. Labeling of hASCs with VSOPs

#### 3.1.1. Detection of VSOPs-Labeled hASCs

**Figure 1:**
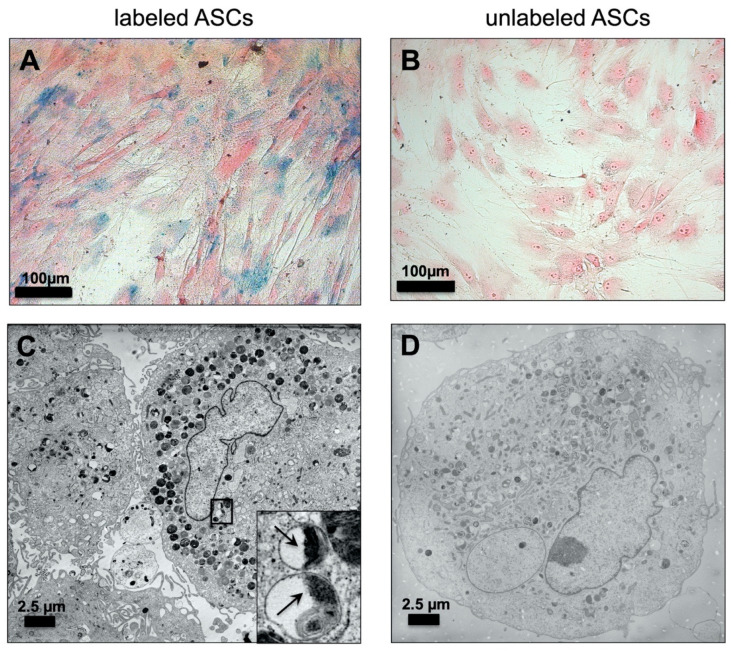
Detection of VSOP-labeled hASCs: Prussian Blue staining of human adipose tissue-derived stromal cells (hASCs) labeled with 1.5 mM citrate-coated very small superparamagnetic iron oxide particles (VSOPs): (**A**) Intracellular blue spots indicate the uptake of iron oxide particles into the hASCs. (**B**) No blue staining was detected in unlabeled controls. Magnification ×200; scale bars represent 100 µm. Transmission electron microscopy (TEM) image analysis: (**C**) Intracellular distribution of vesicles containing VSOPs in hASCs after labeling with 1.5 mM VSOPs. The insert shows individual vesicles at higher magnification with intravesicular VSOPs highlighted by black arrows. (**D**) Within the unlabeled hASCs, no intracellular deposition of VSOPs was detected. Scale bars represent 2.5 µm.

A Prussian Blue staining was used to confirm the VSOPs labeling. Blue staining clearly identified intracellular VSOPs within the labeled hASCs (Figure 1A), whereas no positive staining could be found in unlabeled cells (Figure 1B). In addition, TEM images showed intracellular vesicles with aggregates of VSOPs in the labeled hASCs (Figure 1C). No detectable particles are present in the unlabeled hASCs (Figure 1D).

#### 3.1.2. High-Resolution MRI

**Figure 2:**
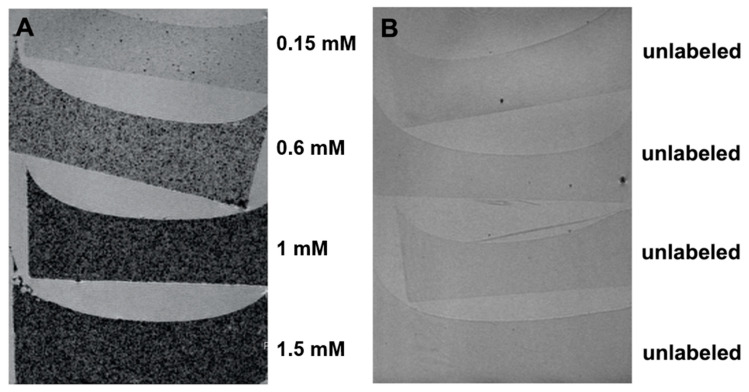
High-resolution MR imaging: (**A**) Magnetic resonance (MR) images of VSOPs-labeled hASCs seeded in agarose gel show the typical hypointense dark spots due to the signal decrease by iron oxide particles. The signal intensity is directly proportional to the concentration of VSOPs used for the labeling procedure. (**B**) The single hypointense spots in the gels with the unlabeled hASCs correspond to microscopic air bubbles located within or at the surfaces of the agarose gels.

VSOPs-labeled cells seeded in agarose gels were detected using high-resolution MR imaging at 11.7 T (Figure 2). The typical signal intensity decrease, presented as hypointense, dark spots, confirmed the presence of VSOPs-labeled cells in the gels (Figure 2A) with lower average signal intensity in the gels with higher VSOPs concentration. Control agarose gel phantoms with unlabeled hASCs showed only single hypointense spots due to microscopic air bubbles (Figure 2B).

### 3.2. Genotoxicity

#### 3.2.1. Comet Assay

**Figure 3:**
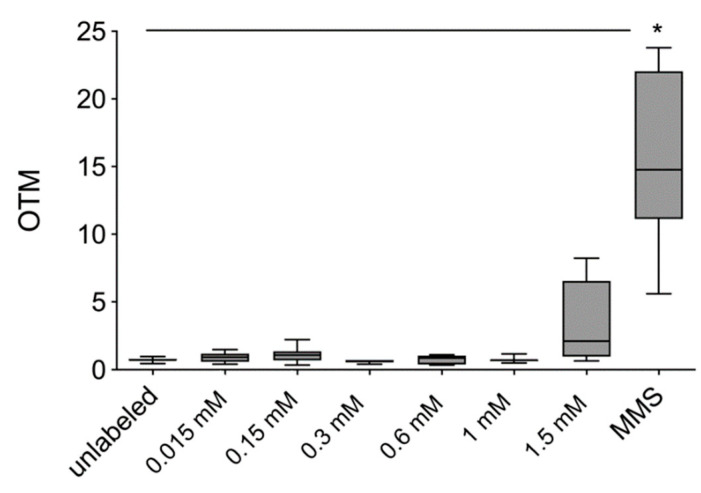
Comet Assay: No significant increase in olive tail moment (OTM) values and thus in DNA fragmentation was observed after labeling of hASCs. A significant increase in OTM values after exposure to 200 µM MMS, which served as a positive control, was determined compared to unlabeled hASCs. Significance is indicated by asterisks (* *p* < 0.0001).

There was no increase in OTM values and thus DNA fragmentation after labeling of hASCs with VSOPs. After exposure to 200 µM MMS (positive control), a significant increase in OTM values was observed compared to unlabeled hASCs. Including the positive control in the statistical analyses, no significant difference in the value of 1.5 mM was determined compared to the control. However, a trend towards an increase in OTM values at 1.5 mM can be seen in the graph (Figure 3).

#### 3.2.2. Chromosomal Aberration (CA) Test

**Figure 4:**
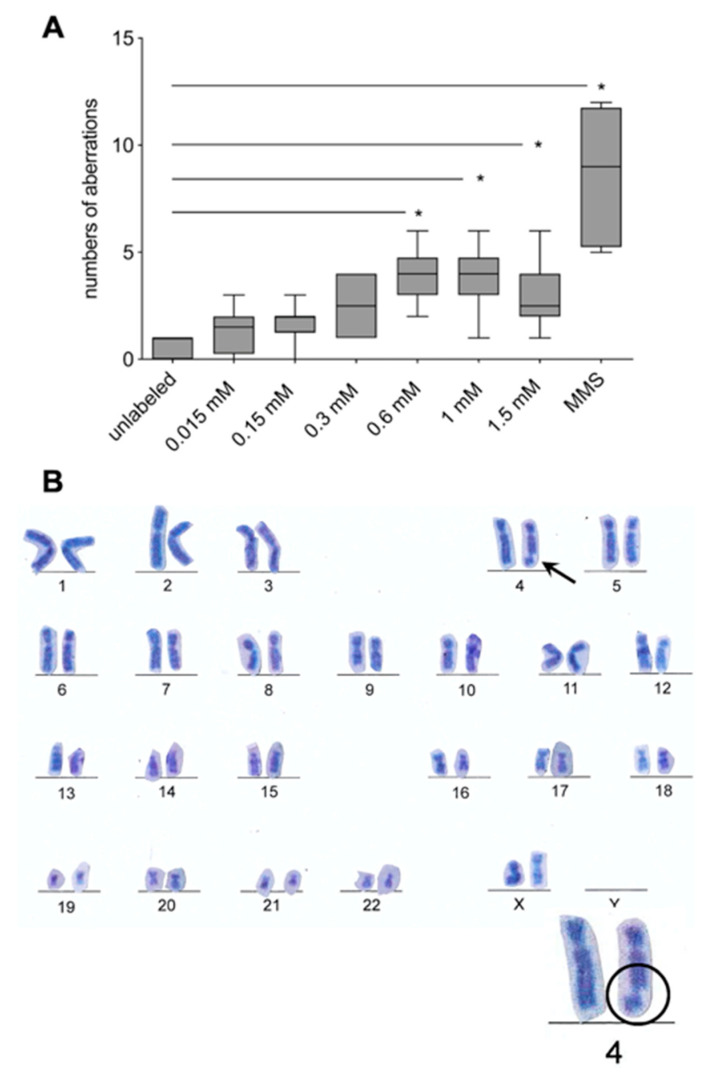
Chromosomal aberration test: (**A**) There was a significant increase in chromosomal aberrations from a concentration of 0.6 mM onwards compared to the unlabeled control (* *p* < 0.001: 0.6 mM, 1 mM; * *p* < 0.05: 1.5 mM). Furthermore, there was a significant increase in chromosomal aberrations after exposure of the hASCs to 200 µM MMS (positive control, * *p* < 0.0001). Significance is indicated by asterisks. (**B**) Chromatid and chromosomal breaks were observed: the example shows a chromatid break of chromosome 4.

A CA test was used to evaluate genotoxicity at the chromosomal level. In the hASCs labeled with VSOPs, there was a significant increase in chromosomal aberrations at a concentration of 0.6 mM or higher compared to the unlabeled control (Figure 4A). Furthermore, there was a significant increase in chromosomal aberrations after exposure of the hASCs to 200 µM MMS. Observed chromosomal aberrations consisted of chromatid and chromosomal breaks (Figure 4B).

### 3.3. Cell Viability and Proliferation Capacity

#### 3.3.1. Trypan Blue Exclusion Test

**Figure 5:**
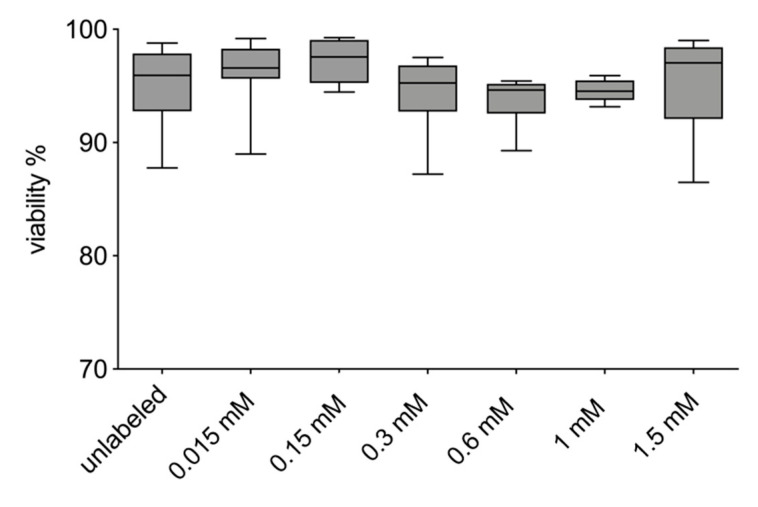
Trypan Blue exclusion test: There was no difference in the viability of the VSOPs-labeled hASCs compared to the unlabeled control.

There was no difference in the viability of the VSOPs-labeled hASCs compared to the unlabeled control. Unlabeled hASCs showed a viability of 95.1% ± 3.6%, VSOPs-labeled hASCs showed a viability of 95.4% ± 4.4%, 94.54% ± 0.96%, 93.82% ± 2.13%, 94.29% ± 3.31%, 97.2% ± 2.0% as well as 96.1% ± 3.1% for 1.5 mM, 1 mM, 0.6 mM, 0.3 mM, 0.15 mM, and 0.015 mM VSOPs (Figure 5).

#### 3.3.2. MTT Assay Proliferation Analyses

**Figure 6:**
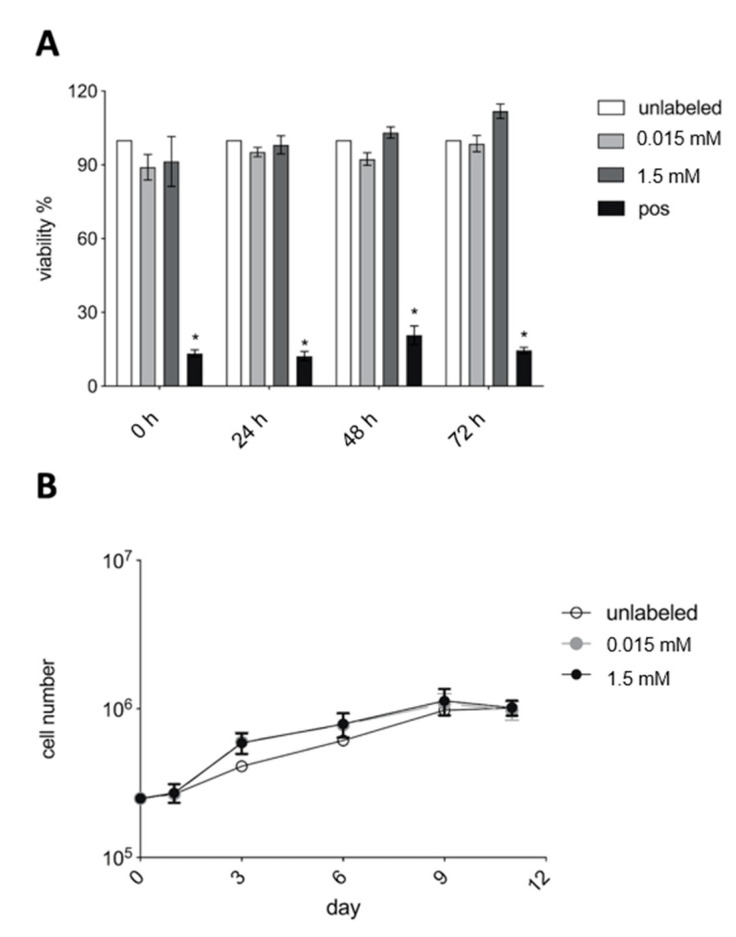
MTT and proliferation assay: (**A**) There was no difference between the viability of hASCs, which were labeled with 0.015 mM (light grey columns) and 1.5 mM VSOPs (dark grey columns), and unlabeled cells (white columns) immediately after the labeling procedure and after 24, 48 and 72 h. The values of the unlabeled cells were normalized to a viability of 100% for each single patient. Mean extinction values for each patient and concentration were normalized to the respective values of unlabeled hASCs from the same patient. Human ASCs treated with tert-butylhydroperoxide (t-BHP) (black columns) showed a significant decrease in cell viability compared to VSOPs-labeled and unlabeled hASCs. Significance is indicated by asterisks (* *p* < 0.0001). (**B**) VSOPs-labeled hASCs showed no difference in proliferation capacity compared to the unlabeled controls.

VSOPs-labeled hASCs did not show decreased viability compared to the unlabeled controls immediately after the labeling procedure and after 24 h, 48 h and 72 h in the MTT assay (Figure 6A). Moreover, VSOPs-labeled hASCs showed no impairment of their proliferation ability over 10 days (Figure 6B).

### 3.4. Scratch Assay for Wound Healing

**Figure 7:**
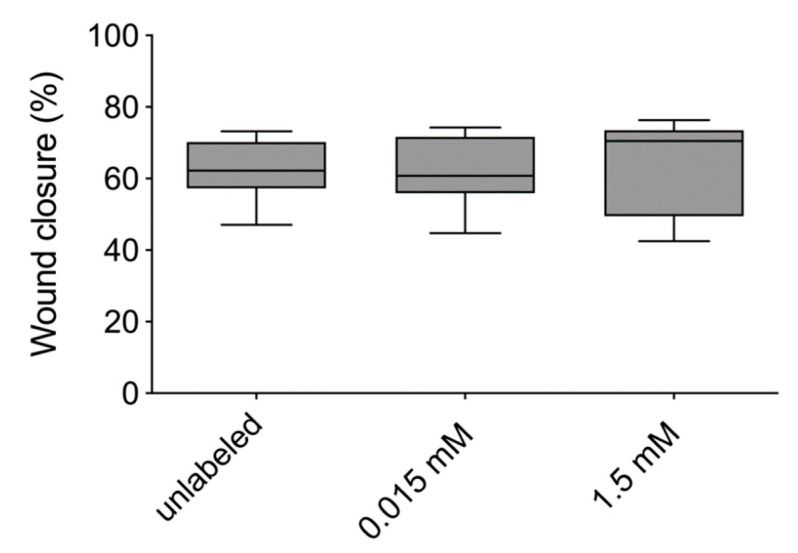
Scratch assay: To evaluate the influence of VSOPs-labeling on the migration ability of hASCs, the scratch assay was used. After 24 h, there was no remarkable difference between labeled and unlabeled cells.

The migration ability of the hASCs was determined with the scratch assay. There was no difference between VSOPs-labeled and unlabeled hASCs (Figure 7). After 24 h, 62.3% of the experimental wound was closed by the unlabeled hASCs and 61.8% by the hASCs labeled with VSOPs at a concentration of 1.5 mM.

### 3.5. Multilineage Differentiation Potential

**Figure 8:**
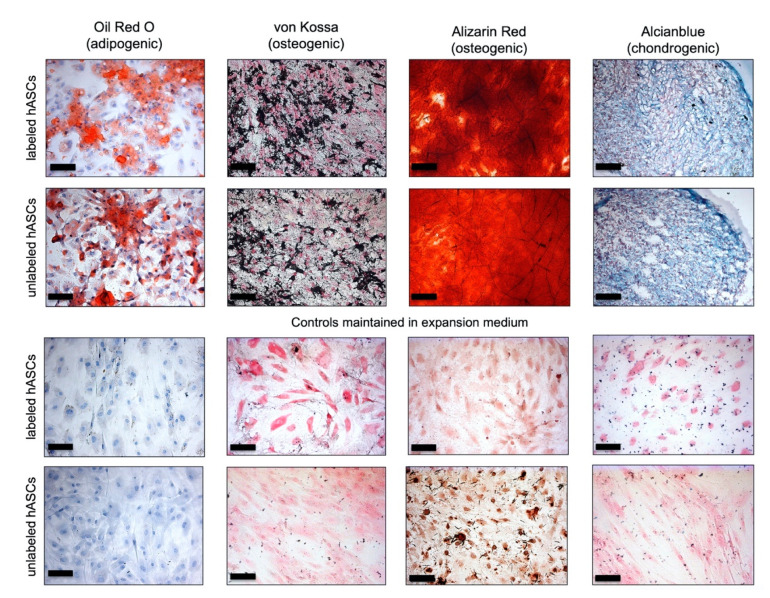
Histological analysis after adipogenic, osteogenic and chondrogenic differentiation: Red-stained, intracellular lipid droplets are apparent in both VSOPs-labeled and unlabeled hASCs after adipogenic differentiation (Row (R) 1 and 2, column (C) 1). The deposition of extracellular calcium was confirmed by the von Kossa stain (C2) and Alizarin Red stain (C3) in both groups. Chondrogenic differentiation was verified by the blue-turquoise staining of acid glycosaminglykans of the extracellular matrix (C4). VSOPs-labeled and unlabeled hASCs, maintained in expansion medium, did not show lipid vacuoles or any extracellular matrix deposition (R3, 4/ C1–4). Magnification ×200; scale bars represent 100 µm in all figures.

There was no difference observed in the histological images after differentiation of VSOPs-labeled and unlabeled cells (Figure 8). Both VSOPs-labeled and unlabeled hASCs showed the typical intracellular lipid droplets after two weeks of adipogenic differentiation using the Oil Red O stain. Labeled and unlabeled hASCs maintained in EM-DMEM did not develop intracellular lipid vacuoles. After three weeks of treatment with osteogenic differentiation medium, VSOPs-labeled hASCs and untreated controls using Alizarin Red and von Kossa staining revealed a deposition of calcified extracellular matrix, which could not be detected in the negative controls. Pellets of labeled and unlabeled hASCs showed the typical blue-turquoise staining of the extracellular matrix after chondrogenic differentiation using the Alcian Blue stain. The control pellets maintained in EM-DMEM showed no blue staining.

**Figure 9:**
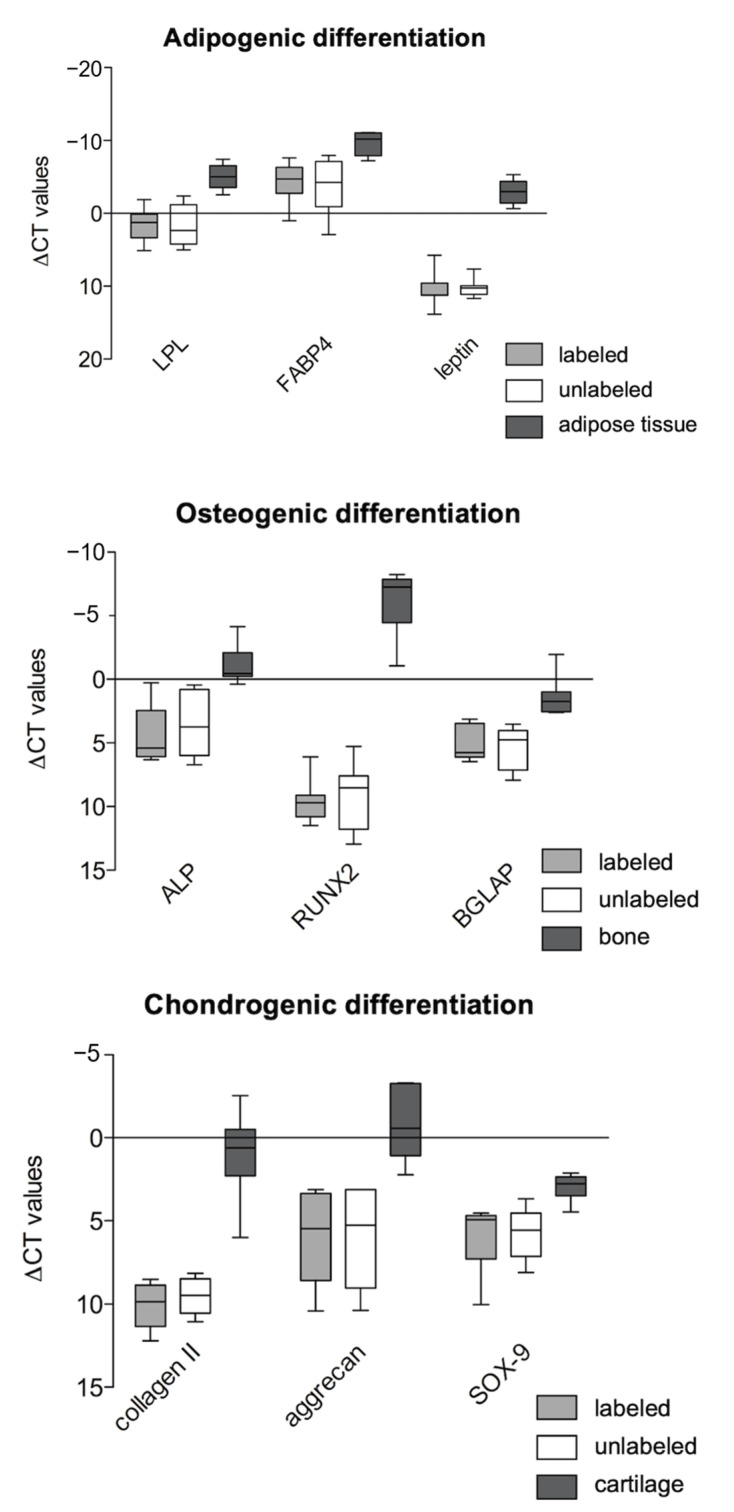
Real time-PCR analyses after adipogenic, osteogenic and chondrogenic differentiation: The presented values (∆CT values) are normalized to the gene expression values of GAPDH. No differences in the expression of specific marker genes after adipogenic, osteogenic and chondrogenic induction between labeled and unlabeled hASCs were detected. Native tissues such as adipose tissue, bone and cartilage were used as positive controls.

Furthermore, the gene expression values of specific adipogenic (FABP4, LPL and leptin), osteogenic (ALP, RUNX-2 and osteocalcin) and chondrogenic (SOX-9, aggrecan and collagen II) marker genes were not different in VSOPs-labeled and unlabeled hASCs (Figure 9).

## 4. Discussion

Iron oxide nanoparticles (IONPs) are used in various biomedical fields. In cancer treatment, they are applied to induce hyperthermia [69,70] or to improve the effectiveness of radiation therapy by generating reactive oxygen species (ROS) [71]. In addition, IONPs can be directed to specific sites in the organism due to their magnetic properties. They are used for tissue engineering [72] and the delivery of biotherapeutic agents [40,70,73,74]. An important diagnostic application of IONPs is the labeling of cells for cell imaging by MRI. However, basic requirements must be met for the labeling procedure: the labeling technique or agent has to be non-toxic and biocompatible. In addition, the agent must not impair the properties, function and behavior of the cells to maintain the effect of the therapy.

In the present study, hASCs were labeled with citrate-coated VSOPs. VSOPs aggregates were found in intracellular, cytoplasmatic vesicles, which was confirmed using the Prussian Blue staining method and TEM image analyses (Figure 1). This is consistent with findings in the literature [31,57]. Agarose gel phantoms, which were seeded with hASCs after labeling with different concentrations of VSOPs, were examined by high-resolution MRI. The detection of hypointense areas increased with the concentration of VSOPs used for the labeling procedure (Figure 2). It has been shown that the effectiveness of labeling depends on the surface/volume ratio of the cells, with a higher probability of endocytotic events and consequently an improved detectability in cells with a higher ratio. Therefore, higher concentrations of IONPs may be necessary for sufficient labeling of certain cells [30,75]. In addition, MRI adjustment protocols play an important role [22,30].

Concentration-dependent negative effects of IONPs, for example on the viability and differentiation potential of labeled cells, have been described and are of high relevance for the use of IONPs in cell-based therapy [26,27,41,43,49,51,52,76]. In the present study, viability was not impaired by the labeling procedure with VSOPs compared to the non-labeled hASCs (Figure 5). In addition, no decrease in viability was observed over the period of 72 h after labeling with different VSOPs concentrations using the MTT assay (Figure 6A). These observations are consistent with the literature, where no reduction in the viability or increased apoptosis of the cells was found after labeling with VSOPs by many authors [9,19,31,38,47,57]. Some authors, however, described concentration-dependent cytotoxic effects of IONPs [43,51,77].

To evaluate genotoxic effects of VSOPs on hASCs, the comet assay was performed [53,56,58,59]. DNA fragmentation degree tends to be increased in the group of hASCs which were labeled with 1.5 mM VSOPs; however, there was no significant difference compared to the unlabeled hASCs and the other VSOPs concentrations (Figure 3). In addition, the chromosomal aberration test demonstrated a significant increase in structural and numerical chromosomal aberrations from a concentration of 0.6 mM (Figure 4). Different statements can be found in the literature regarding the cyto- and especially genotoxicity of IONPs, that may, among others, be due to the use of different cells with different IONPs uptake for the analyses [24,31,42,78,79,80,81,82,83,84]. While IONPs appear to be non-cytotoxic and safe below a concentration of 100 µg/mL, the same study showed evidence of genotoxic effects at concentrations between 10 and 100 µg/mL in the comet assay [25,43,78]. There are no comparable data published that provide information on genotoxic effects of the VSOPs used in the present study. However, the small size of the used VSOPs may be a reason for the genotoxic effects represented by an increased number of chromosomal aberrations in the present study. The induction of DNA strand breaks and DNA damage is supposed to be strongly influenced by the size of the IONPs and their surface coating. Small particles show a greater mutagenic effect compared to larger ones [24,41,82]. The surface area of the nanoparticles, which plays a crucial role in the interaction between nanoparticles and cellular components, increases with the decrease in the particles’ size. Thus, a larger proportion of molecules and atoms of the particles is exposed to the cell structures [41,82]. In addition, the intracellular release of iron ions after VSOPs degradation can lead to an increase in ROS. High, unphysiological levels of ROS induce oxidative stress and lead to protein and lipid peroxidation and DNA damage [20,31,42,85]. ROS-induced imbalances of redox regulation and cellular signaling may also result in genotoxic effects [84,85]. In addition, a direct interaction of the IONPs with the DNA through intercalation of the iron particles within the DNA base pairs has been described [84].

The results of in vitro toxicity tests could overestimate the risk of genotoxicity and tumor formation in vivo after exposition to IONPs [24], but possible consequences such as tumor development cannot be excluded with certainty. Possible toxic implications may be due to local accumulation of IONPs or free iron ions in the tissue. Thereby, a triggered imbalance in the tissue homeostasis can lead to cytotoxicity, DNA damage, epigenetic events, inflammation and oxidative stress. Such mechanisms of iron-induced carcinogenesis and subsequent tumor formation have been described in the literature [42].

To evaluate functional impairment of the hASCs after the labeling, trilineage differentiation procedures were performed. There were no differences observed in the differentiation potential of the labeled and unlabeled hASCs histologically (Figure 8). In addition, using PCR analysis for quantitative assessment both groups showed a positive expression of the specific marker genes after the induction process without significant differences; however, the gene expression values of the cells of the respective native tissue were not reached by the hASCs (Figure 9). The impact of the labeling procedure with IONPs on the differentiation potential of MSCs is discussed controversially [19,22,25,38,46,47,48,50,51,86,87,88,89], especially with regard to their chondrogenic potential, which is described to be impaired in a dose-dependent manner [22,38,45,46,52]. However, a decrease in the adipogenic and osteogenic differentiation potential of MSCs has also been reported [49,51,52]. Differences in particle size, surface coating material, iron load of the cells after labeling as well as the use of transfection agents are supposed to be explanations for the described effects on the functional behavior of different cell types after labeling with IONPs [19,21,22,25,37,38,46,47,48,50,86,87,88]. The results of the present analyses are consistent with several studies [19,30,47,48,86,89], although the study by Heymer et al. [19] is the most comparable, since the same particles and the same differentiation protocols were used. The authors also found no impairment of the adipogenic, osteogenic and chondrogenic differentiation capacity of BMSCs due to the labeling procedure with VSOPs. However, they described in detail a low-grade inhibition of cartilage-specific matrix deposition histologically with an unaffected gene expression of aggrecan and collagen II. Degradation products released by the endosomal VSOPs may lead to enzyme interaction and inhibition of protein synthesis in the cytoplasm [19].

Undisturbed chondrogenic differentiation is contrary to the observations of others, who found an insufficient production of cartilage-specific extracellular matrix after chondrogenic differentiation [22,46]. Andreas et al. [22] assumed on the basis of other studies [20,90,91,92] and their own observations that a high intracellular concentration of IONPs has a negative influence on the organization of the cytoskeleton, especially the actin network, and thus, dose-dependently, inhibits cell functions such as chondrogenesis (mesenchymal condensation), proliferation capacity and cell migration [20,22,90]. However, VSOPs (C-200) did not affect the cytoskeleton, cellular morphology and focal adhesion complexes [20], which may explain the varying results on differentiation potential in the present study and the one of Heymer et al. [19].

The proliferation activity of hASCs was not impaired by the labeling procedure compared to the unlabeled cells in the present study, which is consistent with the results of others, who also used VSOPs (C-200) and MSCs for their studies [9,19]. In addition, no impact on migration capacity was determined. This is in contrast to some authors, who found a decrease in migration capacity and colony formation ability after the labeling of MSCs [37] and neural stem cells [88] with IONPs.

In summary, the present study revealed no cytotoxicity and functional impairment of human ASCs after labeling with citrate-coated VSOPs. An indication of concentration-dependent genotoxic effects is given by the increased number of chromosomal aberrations after the labeling procedure. In the case of in vivo cell monitoring using the MRI in stem cell-based therapeutic approaches, it might be useful to use a higher amount of labeled cells for the application to enhance the detectability in the MRI instead of using higher concentrations of the labeling agent to reduce concentration-dependent effects on the cells.

## Data Availability

Data sharing is not applicable to this article.
